# Structural and Functional Basis of the Fidelity of Nucleotide Selection by *Flavivirus* RNA-Dependent RNA Polymerases

**DOI:** 10.3390/v10020059

**Published:** 2018-01-30

**Authors:** Barbara Selisko, Nicolas Papageorgiou, François Ferron, Bruno Canard

**Affiliations:** CNRS, Aix-Marseille Université, AFMB, UMR 7257, 163 Avenue de Luminy, 13288 Marseille, France; barbara.selisko@afmb.univ-mrs.fr (B.S.); nicolas.papageorgiou@afmb.univ-mrs.fr (N.P.); francois.ferron@afmb.univ-mrs.fr (F.F.)

**Keywords:** RNA virus, RNA genome, *Flavivirus*, RNA polymerase, RNA synthesis, nucleotide inhibitor, selectivity, fidelity, active site, mutation

## Abstract

Viral RNA-dependent RNA polymerases (RdRps) play a central role not only in viral replication, but also in the genetic evolution of viral RNAs. After binding to an RNA template and selecting 5′-triphosphate ribonucleosides, viral RdRps synthesize an RNA copy according to Watson-Crick base-pairing rules. The copy process sometimes deviates from both the base-pairing rules specified by the template and the natural ribose selectivity and, thus, the process is error-prone due to the intrinsic (in)fidelity of viral RdRps. These enzymes share a number of conserved amino-acid sequence strings, called motifs A–G, which can be defined from a structural and functional point-of-view. A co-relation is gradually emerging between mutations in these motifs and viral genome evolution or observed mutation rates. Here, we review our current knowledge on these motifs and their role on the structural and mechanistic basis of the fidelity of nucleotide selection and RNA synthesis by *Flavivirus* RdRps.

## 1. Introduction

### 1.1. Fidelity of RNA Synthesis and RNA Genome Replication

Fidelity of RNA synthesis is an estimation of the frequency of non-Watson-Crick base pairs formed during RNA chain growth driven by RNA polymerases. The same phenomenon is described for DNA polymerases [1,2,3,4,5]. Hence, fidelity of viral RNA synthesis refers to the appearance of non-Watson-Crick base pairs during viral RNA-dependent RNA polymerase (RdRp)-mediated RNA chain growth. Fidelity of viral RNA genome replication is the estimation of the frequency of mutations observed after one, or several, complete virus life cycles have occurred. Here, post-incorporation corrections and the selection of viable genomes are additionally taken into account. Thus, both fidelities (i.e., RNA synthesis fidelity and RNA genome replication fidelity) are related, but not identical [5,6]. They can be quantitated by several numeric indicators ([7], see below).

In this review, we will extend the meaning of fidelity to the process of selecting a correct 5′-triphosphate ribonucleoside (see below). Although incorporation of a modified ribose in a growing RNA strand does not generally produce a mutation in fine, the nucleotide selection process is of utmost importance in the design of nucleoside drug analogues. Fidelity of nucleotide selection is an important part of fidelity of RNA synthesis and, thus, of fidelity of viral RNA genome replication.

In infected cells, viral RdRps have evolved to perform RNA synthesis under fine-tuned conditions. They must synthesize a faithful copy of the viral RNA genome, yet, they have to be able to generate a defined amount of mutations per genome so they can adapt to the adverse response and changing environmental conditions provided by their host cell. Viral RNA genome replication is not a massive, parallel synthesis of similar and independent RNA genomes. Rather, it is best viewed as a process during which a population of interacting or inter-dependent genomes is synthesized. From this population successful mutants are selected exhibiting a selective advantage. Comparison of viral RdRps across species indicates that they are not working at a same fixed mutation rate, or at random, either. A too faithful a synthesis would fail to generate adaptable genomes; a too error-prone an RNA synthesis would lead to a viral population from which no viable genomes would be selected, a so-called “error catastrophe” predicted by Drake [8] and demonstrated by Crotty et al. [9,10]. Structural and functional characteristics of these enzymes (e.g., error rate, processivity, etc.) have evolved for each species specifically. Initially, it was proposed that mutation rates are similar within groups of DNA- or RNA-based organisms when related to the fidelity of genome replication [11,12]. For example, a central value for the mutation rate per replicated genome for RNA viruses was given as 0.76 substitutions per genome per replication [12]. To date with the progress made in nucleic acid sequencing methods, more data is available (although with an implicit high variation [6]), and differences in RNA virus replication fidelity rates emerge. High mutation rates (>1 × 10^−4^ s/n/c = substitutions per nucleotide per cycle of infection) are reported for human *norovirus* G1 (family *Caliciviridae*, genus *Norovirus*) and *Escherichia* virus (bacteriophage) Q-β (*Leviviridae*, *Allolevivirus*) [6,13]. An average value based on several studies [6] of 3.8 × 10^−5^ s/n/c of the mutation rate of hepatitis C virus (HCV, *Flaviviridae*, *Hepacivirus*) genome replication has recently been published. *Coronavirus* Murine hepatitis virus (*Coronaviridae*, *Betacoronavirus*) showed a mutation rate of 3.5 × 10^−6^ s/n/c [6]. Considering the genome size of these viruses, these values correspond to mutation rates per genome and replication of 1.14, 0.59, 0.36, and 0.01, respectively; thus, there is a 100-fold variation.

Measuring fidelity at the RdRp level relies on biochemical assays of recombinant proteins. Misincorporation (i.e., incorporation of the incorrect nucleoside triphosphate (NTP)) may be measured using steady state or pre-steady state measurements [14,15,16,17]. Likewise, the discrimination by the RdRp against non-natural nucleotide analogs (NAs), not only at the base level (non-Watson-Crick base pairs), but also at the ribose level (deoxy-NTP (dNTP) versus NTP), is a measure of RdRp fidelity, [18]. Recently, magnetic-tweezer experiments were developed to study nucleotide analogue incorporation at the single-molecule level [19]. Interestingly, Campagnola et al. [18], using a dNTP/NTP discrimination stop-flow assay, found that fidelity results of this enzymatic RdRp test correlated with deep-sequencing-based mutation frequencies obtained in infected cells. They also found that two RdRps from the *Enterovirus* genus of *Picornaviridae*, Coxsackie virus B3 (CVB3) RdRp and *poliovirus* (PV) RdRp, have different basic fidelities. It is thus reasonable to expect that each *Flavivirus* RdRp has its own characteristic fidelity.

It would be of great importance for many scientific fields (e.g., evolution, vaccine- and drug-design, drug-resistance, epidemiology and prediction of virulence, etc.) to have reliable data of fidelity of RdRp-mediated RNA synthesis, and RNA genome replication. For most viruses, a clear comparative set is not available, and there are almost no data for flaviviruses yet.

### 1.2. Fidelity of Substrate Selection by Viral RdRps

If the nucleotide selection process is altered, synthesis may be terminated or the outcome may have a genetic cost. Mutants may be generated, which are or are not viable in the infected cell. In cells there is a plethora of naturally-occurring nucleotide analogues, which must be selected against in order to produce bona fide viral RNA, and an infectious genome in fine. To achieve selectivity, RNA and DNA polymerases in general have evolved selective “gates” and molecular sensors in their active sites to precisely probe the correctness of the base, the absence or presence of a hydroxyl group at the 2′ position of the ribose moiety of nucleoside triphosphates, and the presence of the triphosphate [2,20,21,22,23]. The need for a correct ribose moiety can be overcome in some instances, though: chemists have proposed ribose modifications to trick viral polymerases. Such NAs that interfere with the RNA synthesis (or DNA synthesis in case of DNA polymerases and reverse transcriptases) constitute the basis of current antiviral therapies against a variety of viruses [24,25]. When they target a viral RdRp, for instance, their mechanism of action is generally expressed through two main effects. First, they can act as RNA chain terminators. Once inserted, incorporation of the next correct nucleotide is impeded. Second, upon incorporation into proviral RNA, synthesis continues normally, but the NA coding capacity is not that of the natural base it mimics. Hence, its presence in the RNA chain leads to a mutation upon subsequent readings as a template. This can finally lead to the above-mentioned error catastrophe. In some instances, NAs may slow or impede RNA synthesis without truly stalling the RdRp, thus combining both effects described above [25,26].

To understand how the fidelity of viral RNA polymerases is achieved has implications on our understanding of not only viral mutagenesis and evolution, but also on the clever design of NA drugs to control viral infections.

### 1.3. RdRp Fidelity within the Context of the General Scheme of the Polymerization Reaction

Nucleic acid polymerases create a phosphodiester bond between the 3′-OH and the 5′-α-phosphate of two nucleotides, in a nucleic acid template-dependent or independent manner. RdRps depend on an RNA template. Generally, the 5′-α-phosphate belongs to a 5′-triphosphate nucleotide, and receives a nucleophilic attack from either the 3′-OH of a single nucleotide or that of a short oligonucleotide primer or, in some cases, an -OH group of a peptide or protein primer.

**Figure 1:**
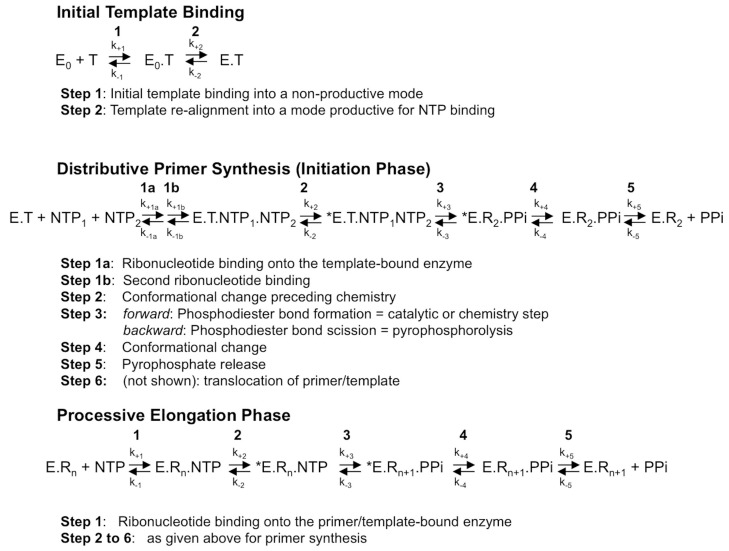
The mechanism of RNA synthesis by a viral de novo initiating RdRp. Although primer synthesis can be template-independent in flaviviruses, the current general model for de novo RNA synthesis by viral RdRp including *Flavivirus* RdRp is best described with three distinct phases, that of template binding into a productive mode (upper scheme), followed by primer synthesis (here, a di-ribonucleotide, middle scheme) and primer elongation (lower scheme). The constants k_+1_ to k_+5_ and k_−1_ to k_−5_ represent the forward and backward kinetic constants of each individual reversible reaction, respectively. The constants k_+1_ to k_+5_ and k_−1_ to k_−5_ for each phase are not equivalent, as they do not represent the same reactions. T, template; E_0_, free enzyme; E, enzyme bound to the template under a productive mode for nucleotide binding; *E, catalytically competent enzyme for nucleotide incorporation; NTP, correct (i.e., template-complementary) ribonucleotide 5′-triphosphate; R_n_, RNA made of the primer of n nucleosides annealed to the template. Only the dinucleotide primer synthesis step is shown, primer synthesis may comprise more steps; PPi: pyrophosphate.

RdRps from some viral families (e.g., *Flaviviridae*, *Coronaviridae*, *Cystoviridae*, *Leviviridae*) are able to synthesize their own oligonucleotide primer and, hence, start nucleic acid synthesis de novo. The corresponding first phase of RNA synthesis is named “initiation”. Initiation is a distributive polymerization reaction, i.e., the enzyme dissociates frequently from the template. The next phase consists in a processive RNA synthesis reaction, called “elongation”. Figure 1 summarizes the current step-wise model for both phases. They share a five-step kinetic reaction scheme for nucleotide incorporation (after the binding of the RdRp to the template or primer/template): (1) the binding of the NTP; (2) a conformational change to perfectly align the NTP for incorporation; (3) the catalytic step itself: forward—the metal-assisted phosphodiester bond formation; backward—phosphodiester bond scission by pyrophosphate, called pyrophosphorolysis; (4) reverse conformational change; (5) the release of the byproduct pyrophosphate; and, finally, (6) the translocation along the template.

**Figure 2:**
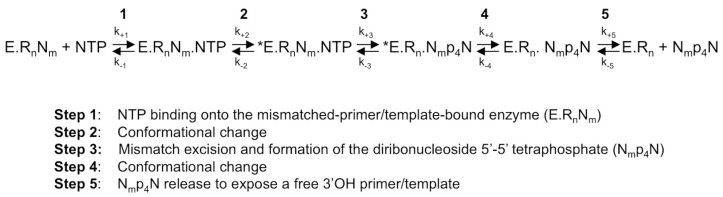
The NTP-mediated nucleotide excision pathway by HCV RdRp [30]. A mismatched 3′-end nucleotide is excised by a pyrophosphorolysis-like reaction, in which the pyrophosphate donor comes from an undefined NTP of the intracellular nucleotide pool. E, enzyme; R_n_N_m_: primer annealed to the template with a 3′-terminal mismatch; R_n_: perfectly matched doubled-stranded RNA; *E: catalytically competent enzyme; NTP: correct (i.e., template-complementary) NTP; N_m_p_4_N: diribonucleoside 5′-5′ tetraphosphate made of the 3′-base of mismatched nucleoside N_m_ linked by a 5′-tetraphosphate-5′ bridge to a nucleoside coming from the intracellular nucleotide pool.

First of all, there are numerous indications that step 2, the first conformational change where the probing and accommodation of the incoming NTP is taking place, is important for RdRp fidelity. These will be further described below. Step 2 should influence the efficiency of step 3, the nucleotide or analogue incorporation, which is important for fidelity especially for the interference of correction mechanisms. Indeed, having the reverse reaction (pyrophosphorolysis) occurring much more efficiently on a mismatched base than on the regular Watson-Crick base, this reverse process represents de facto a correction mechanism operating to increase the overall nucleotide insertion fidelity. In reality, this process is very likely to occur since the time of residency of a mismatch in the RdRp active site is expected to be significantly longer than that of a regular base. A mismatch produces a stalling of the RdRp, whereas a Watson-Crick nucleotide is immediately extended by the next correct nucleotide. Interestingly, the pyrophosphate level in the cell is in the micromolar range and stringently controlled by cellular pyrophosphatases, whereas the NTP level in the cell is higher, in the millimolar range [27,28]. Additionally, the γ- and β-phosphates of NTPs are, structurally, almost identical to a pyrophosphate molecule, and they can catalyze the elimination of the terminal nucleotide in a reaction analogous to pyrophosphorolysis, resulting in a primer shorter by one nucleotide and the concomitant production of a nucleoside tetraphosphate NppppN (Figure 2). This repair mechanism turned out to be the main mechanism of resistance against the NA azidothymidine (AZT) mediated by the reverse transcriptase (RT) of HIV [29]. In the case of HCV RdRp, Jin et al. [30] have reported that this NTP-mediated mismatch-removal activity is indeed a mechanism of fidelity of RNA synthesis through excision-repair. It is not known how widespread this mechanism is in the case of other viral RdRps.

Since the general reaction mechanism of nucleotide addition is shared by all polymerases, the active site of viral RdRps is made of catalytic motifs with a conserved secondary structure, motifs A and C, and two aspartic acid residues common to all polymerases. Another two motifs, B and D, are common to RNA-dependent polymerases [31]. Motifs F and E are shared by RdRps and reverse transcriptases [32]. Finally, motif G has first been identified for primer-dependent RdRps [33] but is structurally conserved in de novo RdRps (see below). Most of these motifs not only play a decisive role in the general nucleotide addition scheme, but there is also evidence that they are implicated in RdRp fidelity, as outlined below. The aim of this review is to summarize the structural and functional information on the involvement of motifs A to G in the fidelity fine-tuning of viral RdRps, and relate them to the current knowledge on *Flavivirus* RdRps.

## 2. *Flavivirus* RdRps 

De novo viral RdRps, and *Flavivirus* RdRps in particular, have evolved features that are usually accomplished by ancillary proteins in the eukaryotic cell. They synthesize their own primer at the precise 3′-end of the (+) genomic and (−) anti-genomic viral RNA, and leave the (−) anti-genomic RNA 5′-end uncapped, but cap the genomic (+) RNA 5′-end. The de novo RdRp function of flaviviruses is harbored in the C-terminal domain (RdRp domain 74 kDa) of protein NS5, which also contains an N-terminal domain with methyltransferase (MTase domain 30 kDa) functions involved in RNA cap formation [34].

### 2.1. Structures

The first crystal structure of a nucleic acid polymerase (DNA polymerase, Klenow fragment) in 1985 [35] established a presently widely accepted analogy to a cupped right hand, with fingers, palm, and thumb subdomains. The first complete structure of an RdRp, HCV RdRp determined in 1999 [36,37,38], showed for the first time that a characteristic feature of viral RdRps is the connection between the finger and thumb subdomains by two loops (the so-called fingertips of index and ring finger), which reduce the overall flexibility of the RdRp. The active site is enclosed and instead of large subdomain movements, as it has been shown for the Klenow fragment and other polymerases [39], the conformational changes of RdRps upon substrate probing and accommodation and reaction co-product release are rather subtle. This has been demonstrated in a series of highly interesting ternary complex structures of several primer-dependent *Picornaviridae* RdRps, notably of foot-and-mouth disease virus (FMDV, genus *Aphtovirus*, [4]), PV [40], and *enterovirus* 71 (EV71) [41]. These movements are also visible by comparing other apo- or pre-catalytic complex structures with ternary complex structures in a catalytic state as for HCV RdRp (PDB ID: 2XI3 [42]) versus complex structures (PDB ID: 4WTA [43]). They will be discussed in detail below.

**Figure 3:**
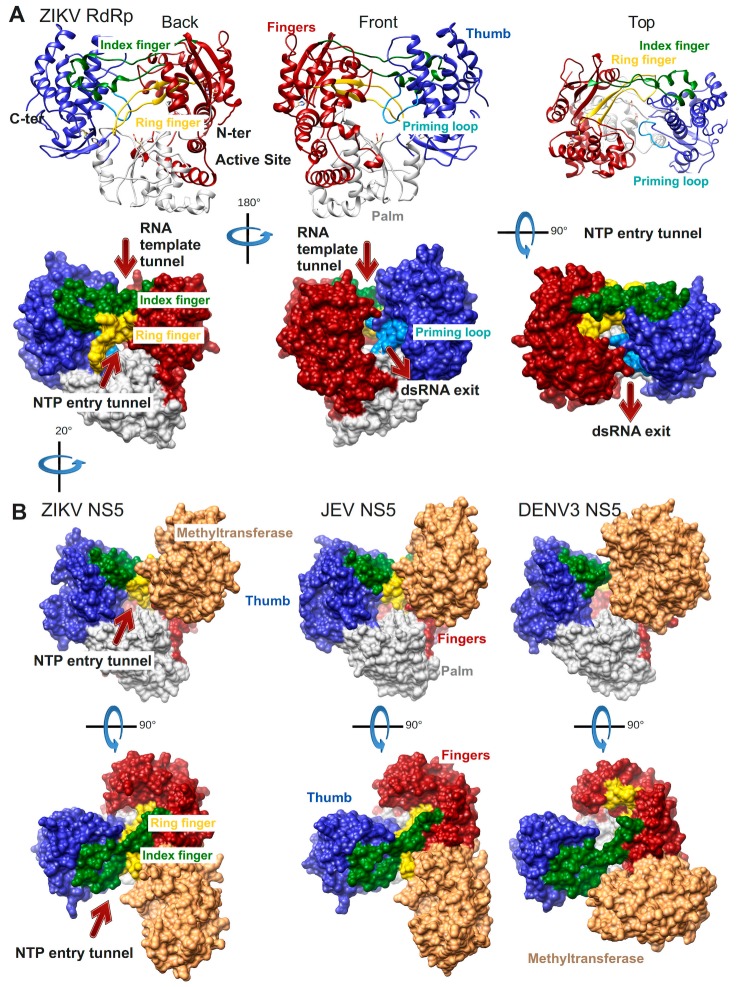
*Flavivirus* RdRp structures. (**A**) Structure of the ZIKV RdRp domain (PDB ID: 5U0C) in ribbon representations above and surface representations below. Back, front, and top views are shown. Important structural and functional features are colored (palm subdomain, gray; fingers subdomain, dark red (index finger, dark green and ring finger, gold); thumb subdomain, medium blue (priming loop, light blue)) and/or labeled. The side chains of the active site residues D335 (motif A) and D666 (motif C) are shown in sticks. (**B**) Structures of ZIKV full-length NS5 (PDB ID: 5U0B), JEV NS5 (PDB ID: 4K6M), and DENV3 NS5 (PDB ID: 4V0Q) containing the RdRp and methyltransferase (MTase in light brown) domains in surface representation using the same color code as in (**A**). Back views are rotated by ca. 20° to the right in relation to the representation in (**A**) in order to show the NTP entry, which is partially obstructed by the MTase domain.

The first *Flavivirus* RdRp structure was determined in 2007 and was of the isolated RdRp domain of West Nile virus (WNV, PDB ID: 2HFZ [44]). Since then more RdRp domain structures have been resolved as apo enzymes or in complex with NTP or inhibitor molecules: dengue virus serotype 3 (DENV3, PDB ID: 2J7W, 2J7U [45], PDB ID: 4C11 [46] and several inhibitor complex structures [47,48,49]), DENV2 (PDB ID: 5K5M [48]), Japanese encephalitis virus (JEV, PDB ID: 4MTP, 4HDG, 4HDH [50]) and most recently Zika virus (ZIKV, PDB ID: 5U04 [51], PDB ID: 5WZ3 [52], PDB ID: 5U0C [53]). The general features of *Flavivirus* RdRps are illustrated in Figure 3A using the most complete of the recently published structures of ZIKV RdRp (PDB ID: 5U0C): the subdomains fingers, thumb, and palm of the cupped right hand structure, the template tunnel, the priming loop pointing from the thumb subdomain towards the active site (more details below), the active site being harbored by the palm domain, the dsRNA exit tunnel, the NTP entry tunnel and the two fingertip loops (of index and ring finger) connecting the fingers and thumb subdomains.

The structures of three full-length NS5 proteins have been determined so far: of JEV (PDB ID: 4K6M [54]), DENV3 (PDB ID: 4V0Q [55], PDB 5CCW [56]) and ZIKV (PDB ID: 5TFR [57], PDB 5U0B [43]) (Figure 3B). Two of them, JEV and ZIKV NS5, present an identical interface between the RdRp and the MTase domains whereas DENV3 NS5 adopts a different interface. In all three cases though the MTase domain is localized near the NTP entry tunnel of the RdRp domain. The residues of both interfaces have been shown to be functionally relevant for viral replication (JEV [58], DENV3 [55]). It is not yet clear during which step of RNA synthesis these interfaces are relevant. The RdRp domain in ZIKV NS5 (PDB ID: 5U0B) does not show major conformational changes compared to the isolated RdRp domain (PDB ID: 5U0C). There is a local change in the conformation of the NTP tunnel ceiling constituted by the ring finger (compare the backview of ZIKV RdRp in Figure 3A left side to the back view just below of ZIKV NS5 in Figure 3B). This change seems to be due to the presence of the MTase domain, which contacts the tip of the ring finger (for more details refer to the Motif F section below).

### 2.2. Functional Characteristics

As stated above, *Flavivirus* RdRps are able to synthesize RNA without the help of a primer. The *Flavivirus* NS5 binds to specific viral RNA sequences (and avoids other spurious cellular RNA 3′-ends) [59,60], and synthesizes a di-ribonucleotide primer complementary to the exact 3′-end genomic sequence [61]. The enzyme has evolved to be able to synthesize the exact pppAG di-ribonucleotide, which is conserved in the *Flavivirus* genus, under a variety of conditions of template sequence, template presence/absence, and metal ions in vitro. The structural element decisive for de novo initiation is the priming loop that has been observed in the first *Flavivirus* RdRp structures [44,45]. Later it was demonstrated by mutational and functional analysis that it is indeed essential for de novo initiation. A strictly conserved His is the strongest candidate for forming a priming platform against which the first nucleotide stacks upon de novo initiation [61]. The strict specificity for ATP as the priming nucleotide points to the existence of a specific ATP-binding site involving the priming loop. Structurally, this site has not yet been characterized because of the lack of relevant binary or ternary complex structures of *Flavivirus* RdRps. Interestingly, the loop can be deleted without major impeachment of RNA synthesis. The resulting enzyme is now able to initiate RNA synthesis at various internal sites of an RNA template. Accordingly, the structure of a ternary complex enzyme:primer/template:nucleotide of HCV RdRp in the catalytic state [43] was obtained using an enzyme construct from which the corresponding priming loop had been deleted. This also indicates that the loop clears off the exit RNA pathway without major distortion nor dysfunction of the RdRp active site. Thus, the enzyme switches with a conformational change including the opening of the priming loop from a distributive mode to a processive elongation mode of RNA synthesis. This change in enzyme behavior and properties is also still very poorly characterized. It can be observed during kinetic assays [62], but the associated structural changes are still elusive.

Since *Flavivirus* RdRp structures are flexible, it has been difficult to set up test systems such as transient kinetic systems with a stable elongation complex as it has been possible for PV RdRp [63,64]. It is thus not yet known which step in the reaction scheme given in Figure 1 is rate-limiting, nor has it been shown biochemically that a conformational change before the elongation reaction really happens. The only published study of a stable, highly-processive elongation complex used the DENV2 RdRp domain [16]. The authors studied nucleotide discrimination (i.e., the ratio of the incorporation efficiency of correct GTP versus incorrect NTP) by DENV2 RdRp, which was 34,000 to 1 for UTP, 59,000 to 1 for ATP, and 135,000 to 1 for CTP. Interestingly, for HCV RdRp GTP discrimination was reported to be 14,500 to 1 for UTP, 450,000 to 1 for ATP and 42,000 to 1 for CTP [65]. More studies like these are desirable but await stable and highly-processive elongation complexes of full-length *Flavivirus* NS5 with a primer/template [17,54,66].

## 3. Structural Motifs and Other Putative Fidelity Checkpoints

In this section, we discuss in detail *Flavivirus* RdRp motifs A to G and their proven or potential involvement in RNA synthesis fidelity by relating them to existing fidelity data. There are only very scarce data for flaviviruses regarding RdRp variants of nucleotide selection fidelity. Few variants and associated data have been reported for WNV [17] and for St. Louis encephalitis virus (SLEV) [67]. When the search is extended to the *Flaviviridae* family, there is no large increase of the number of fidelity variants reported in the literature either. Most studies relate to nucleoside-analogue-resistant variants mapping to the HCV RdRp gene [68,69]. Here, we make use of the wealth of fidelity data accumulated particularly in the *Picornaviridae* family to benefit the *Flavivirus* RdRp field.

**Figure 4:**
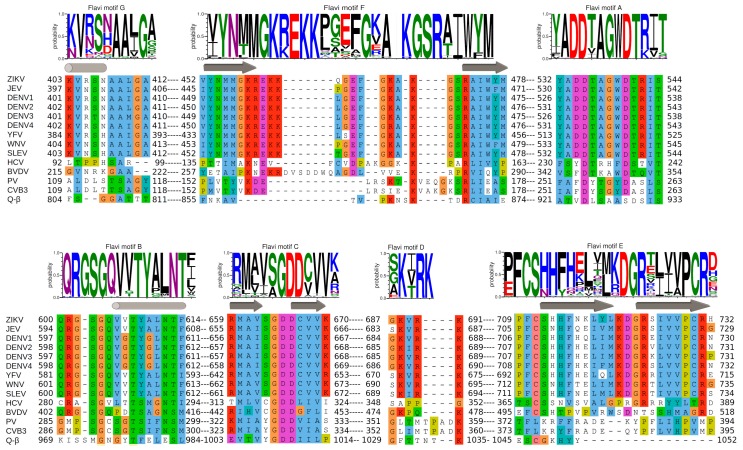
Conservation of sequence and structural motifs A to G within *Flavivirus* RdRps (Weblogo) and selected positive-strand RNA virus RdRps (alignments). Motifs are given as they appear; N- to C-terminus in the primary sequence of the RdRps: G, F, A, B, C, D, E. Selected sequences of positive-strand RNA virus RdRps are from: ZIKV (PDB ID: 5U0C), JEV (PDB ID: 4K6M), DENV1 (NCBI NP_722465), DENV2 (PDB ID: 5K5M), DENV3 (PDB ID: 5CCV), DENV4 (NCBI NP_740325), YFV (NCBI NP_041726), WNV (NCBI NP_041724), St. Louis encephalitis virus (SLEV, NCBI YP_001008348), HCV (PDB ID: 4WTA), bovine viral diarrhea virus (BVDV, PDB ID: 1S49), PV (PDB ID: 3OL6), Coxsackie virus B3 (CVB3, PDB ID: 4ZPD), and bacteriophage Q-β (PDB ID: 3AVT). The alignment was based on the structural alignment of the RdRps provided by Olve Peersen for the present series of reviews. Manual adjustments were done within motifs F and D. Numbers correspond to the first and last residue in the given motif. The alignment representation was generated using Jalview [71], which applies ClustalX color codes. The secondary structure of ZIKV RdRp (PDB ID: 5U0C) is given above the sequence alignment. For the Weblogo part, the RefSeq protein database of NCBI was searched for “NS5 *Flaviviridae*”; 123 sequences were recovered of which repeated sequences were removed. Of the resulting 82 *Flavivirus* sequences, sequences with unknown residues were removed. Finally, the amino acid conservation within the motifs of 79 unique *Flavivirus* RdRp sequences was analyzed using Jalview [71] and represented with Weblogo [70]. The color codes given byWeblogo consider the chemical characteristics of each amino acid.

In Figure 4 we show a WebLogo analysis [70] of sequence conservation within motifs A to G of 79 *Flavivirus* RdRps (ordered according to their N- to C-terminus occurrence within the primary sequence: G, F, A, B, C, D, E). Below each WebLogo a combined structure- and sequence-based alignment of the corresponding motifs A to G is placed, including a number of relevant flaviviruses (see the figure legend) in comparison to other positive single-strand RNA virus RdRps for which fidelity data exist (HCV, bovine viral diarrhea virus (BVDV) belonging to *Flaviviridae*; PV, CVB3 from *Picornaviridae*; *Escherichia* virus Q-β from *Leviviridae*). The motifs are sequentially presented in the accompanying movies, which also show a model of the detailed pathway of nucleotide incorporation into RNA by ZIKV RdRp (Video S1 and S2).

### 3.1. Motifs A and C

**Figure 5:**
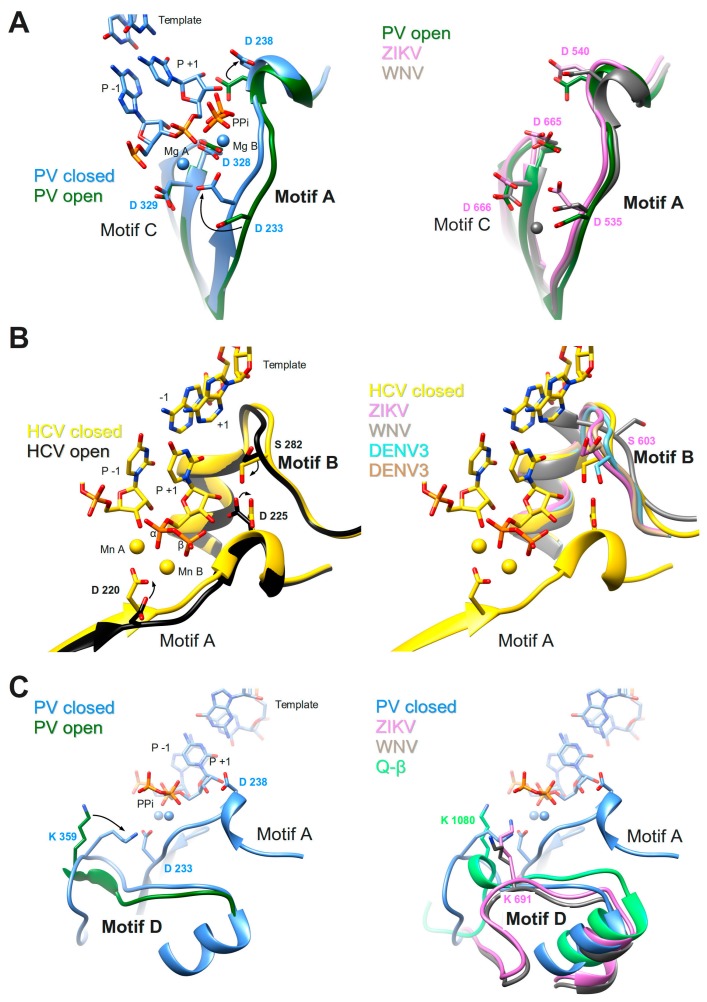
Conformational changes of motifs A, B, and D of PV RdRp and/or HCV RdRp upon reaction (left panels) in comparison to motif conformations of *Flavivirus* RdRps (right panels). (**A**) Motif A; left panel: ribbon representation of PV RdRp (PV closed, PDB ID: 3OL7, blue) in complex with template (only the base part of −1 and +1 shown) and product (only P−1 and P+1 shown) represented in sticks (colored according to atom type: C, blue; O, red; P, orange), and two Mg^2+^ ions as blue spheres, just after the incorporation of P+1. The liberated pyrophosphate (PPi) is still present at the catalytic site. PV open (PDB ID: 3OL6, dark green) corresponds to a complex after reaction and translocation. Only the protein is shown. Selected amino acid side chains are shown and colored according to atom type. Conformational changes are indicated by black arrows. Right panel: PV open as in the left panel superimposed to ZIKV RdRp (PDB ID: 5UOC, orchid) and WNV RdRp (PDB ID: 2HFZ, grey). The Mg^2+^ ion (grey) belongs to the WNV RdRp structure. (**B**) Motif B; left panel: ribbon representation of HCV RdRp (HCV closed, PDB ID: 4WTA, gold) in complex with template (only −1 and +1 nucleobases are visible), product (only P−1 shown), and incoming UDP (P+1) represented in sticks (colored according to atom type: C, gold; O, red; P, orange), and two Mn^2+^ ions as golden spheres. HCV open (PDB ID: 2XI2, black) corresponds to the apo enzyme. Selected amino acid side chains are shown and colored according to atom type. Conformational changes are indicated by black arrows. Right panel: HCV closed, as in the left panel, superimposed to ZIKV RdRp (PDB ID: 5UOC, orchid), WNV RdRp (PDB ID: 2HFZ, grey), DENV3 NS5 (PDB ID: 4V0Q, light blue) and DENV3 NS5 (PDB ID: 5CCV, brown). (**C**) Motif D; left panel: PV closed (PDB ID: 3OL7, blue) with template, product, and PPi (see explanation in (**A**). A larger protein stretch than only motif D is shown including the helix before and the loop after motif D. For PV open (PDB ID: 3OL6, dark green) only the protein region is shown that corresponds to motif D as given in Figure 4. Selected amino acid side chains are shown and colored according to atom type. A conformational change of the loop and residue K359 is indicated by a black arrow. Right panel: larger motif-D stretch of PV closed shown with template, product, and PPi as in the left panel, and superimposed to ZIKV RdRp (PDB ID: 5UOC, orchid), WNV RdRp (PDB ID: 2HFZ, grey) and bacteriophage Q-β RdRp (PDB ID: 3AVT, green). Selected amino acid side chains are shown and colored according to atom type.

These two motifs constitute the core of the catalytic site where the chemical reaction of phosphoryl transfer occurs. The overall structure is made of two β-strands connected by a short loop (Motif C) and a β-strand followed by a short helix (Motif A). Two aspartate residues coming from each motif, are universally conserved in all polymerases. In plus-strand virus RdRps the motif A signature is DxxxxD. Additionally, there is a conserved aromatic/hydrophobic residue at the beginning and a hydrophobic residue followed by S/T at the end of the motif (alignment, Figure 4). Motif C shows a GDD signature preceded and followed by hydrophobic residues forming the β-strands. As seen in the crystal structures of PV RdRp [40] and EV71 RdRp [41], after binding or translocation of the primer/template RNA, the next incoming nucleotide is first probed by base pairing and its final perfect alignment for reaction includes a movement of catalytic motif A towards the motif C completing a three-stranded β-sheet (Figure 5A left panel comparison of open and closed form of PV RdRp (PDB IDs 3OL6 and 3OL7, respectively)). There is a side chain movement (“out” to “in”) of the catalytic aspartic acid located in motif A enabling the exact positioning of Mg^2+^ ion A. The ion together with the second Mg^2+^ ion B, brought in by the NTP, promotes the nucleophilic attack of the 3′-OH of the primer onto the α-phosphate of the incoming nucleotide. The movement of motif A includes also the change of the side chain position of the second aspartic acid within the motif (movement “in” to “out”) leaving space for the 2′OH group of the aligned NTP. The proposal that the conformational change of motif A represents a fidelity checkpoint is supported by studies using *Picornaviridae* RdRps where mutated hydrophobic residues within motif A showed changes in fidelity (FMDV RdRp [72], and CVB3 RdRp [18]). The same kind of movement of motif A is observed for the de novo RdRp of HCV (apo structure PDB ID: 2XI2 [42] versus complex (PDB ID: 4WTA, [43]) (see below).

In *Flavivirus* RdRps 8 of 13 residues of motif A (xADDxAGWDTxxx) are strictly conserved (WebLogo Figure 4). Conservation of motif C is less extensive but the GDD signature and two Val residues are conserved throughout. When analyzing the *Flavivirus* RdRp structures superimposed to the closed and open conformations of PV RdRps (using the superimposed set of viral RdRp structures accompanying this special issue on viral RdRps provided by Olve Peersen), it is obvious that all *Flavivirus* RdRp structures contain motif A in an open conformation; the three-stranded β-sheet of motifs A and C is not formed. This is illustrated in the right panel of Figure 5A by comparing the open form of PV RdRp (PDB ID: 3OL6) with ZIKV RdRp (PDB ID: 5U0C) and WNV RdRp (PDB ID: 2HFZ). Whereas catalytic residue D535 in ZIKV RdRp points towards the catalytic center but needs adjustment to reach the catalytic position, the corresponding WNV RdRp residue is in the “out” position and fixes an Mg^2+^ ion in a pre-catalytic position that has also been seen in the open form of EV71 RdRp complex structure (PDB ID: 5F8I, [41]). Motif-A residue D540 (ZIKV RdRp numbering) is in the “in” position, also corresponding to the open conformation of PV and HCV RdRps (see summary in Table 1). We, thus, surmise that as for PV and HCV RdRp, one fidelity gate for *Flavivirus* RdRps is the formation of the motif-C-motif-A β-sheet. The pre-catalytic Mg^2+^ ions present in the WNV and EV71 RdRp structures seem to show two distinct positions for metal ion A on its way into the catalytic site. This suggests a movement of metal ion A from “out” to “in” which would contribute to the formation of the β-sheet and the closure of the catalytic site.

*Flavivirus* RdRp motif-A mutations having an impact on nucleotide selectivity are currently unknown. Putative fidelity-changing residues that correspond to the residues of motif A from the mutational studies of *Picornaviridae* RdRps are Y532, D534, T541 (ZIKV RdRp numbering), the latter are strictly conserved in *Flavivirus* RdRps (Weblogo Figure 4). Interestingly, in contrast to *Picornaviridae* and *Flaviviridae* RdRps which seem to use the closure of motif A as a fidelity checkpoint, the structure of low-fidelity bacteriophage Q-β RdRp shows a preformed motif-C-motif-A β-sheet with catalytic D968 side chain in place in the apo structure (included in Table 1).

### 3.2. Motif B

Motif B consists in a loop followed by an α-helix. The conserved signature is SGxxxTxxxN at the sequence level of plus-strand RdRps (alignment Figure 4). The loop was observed to be highly flexible, especially in apo RdRp structures (reviewed in [73]). A wide-range closing and opening movement is proposed to be involved in the translocation step after catalysis. Diagnostic probes for the movement of motif-B loop during catalysis were presented [74]. The final adjustment of the loop after translocation and accommodation of the next incoming nucleotide seems to imply rather limited movements, observed again for *Picornaviridae* and HCV RdRp (apo enzyme (PDB ID: 2XI2, [42]) versus complex (PDB ID: 4WTA, [43] shown in Figure 5B left panel). The adjustment of the loop (main chain flip of the conserved Gly (G283 in HCV) and the side chain move of a conserved Ser (S282) residue) allows the proper positioning of the ribose moiety of the NTP via the establishment of a hydrogen bond network towards the 2′OH group of the ribose. The hydrogen bond between the Ser side chain and the 2′O atom closes the active site in concert with the move of the motif-A Asp (D225 in HCV) away from the 2′OH group discussed above (see also Table 1). In contrast to the flexibility of the loop, the α-helix part of motif B remains in place and especially the strictly conserved Asn at the end of motif B (not shown in Figure 5B) contributing to the hydrogen-bond network to the 2′-OH group of the ribose. The conformational change of motif B is considered another fidelity check point of viral RdRps [64]. Interestingly, the Ser of motif B is replaced by a Met in low fidelity Q-β RdRp (Table 1).

*Flavivirus* RdRps show important sequence conservation in motif B with an almost strictly conserved QRGSGQVxTYxLNTx signature (WebLogo Figure 4). The conformation of the *Flavivirus* RdRp motif B loop in the existing structures varies widely with the S603 (ZIKV numbering) side chain mainly adopting the “out” position as in the open conformation of PV or HCV RdRps or a displaced “in” position (right panel Figure 5B and Table 1). A special conformation is adopted in the WNV RdRp (PDNB ID: 2HFZ) where the loop might show a translocation conformation closing in towards template positions −1 and +1 whereas the Ser seems to be totally displaced (Figure 5B right panel). Another motif-B residue, conserved in *Flavivirus* RdRps as an Arg (R601 in ZIKV) corresponds to M296 of FMDV RdRp which has been shown to be a fidelity determinant [75]. An equally conserved Thr (T613) corresponds to S299, important for fidelity of CVB3 RdRp [18].

Residues located in the vicinity of motif B and probably influencing its conformational flexibility have been reported to be fidelity modulators. PV RdRp residue H273 is located in a β-sheet in the fingers domain behind motif B [18,76]. This β-sheet is present in *Flavivirus* RdRps with corresponding residue V579 (ZIKV). Another is CVB3 RdRp residue Y268 [18] at the end of the helix situated just before the H273 β-strand. The corresponding residue in ZIKV RdRp is Y576. Finally, Yang et al. [74] have reported that changes occurring greater than 20 Å apart, such as a G64S mutation situated near the essential N-terminus of PV RdRp, may result in structural and/or dynamic changes of motif B. They suggest that a long-distance network transduces changes in ligand binding, conformational changes, and catalysis, affecting polymerase fidelity in fine.

Due to the success of 2′-modified nucleoside analogues as *Flaviviridae* inhibitors, amino acids have been identified that are directly involved in resistance to said analogues [68,69]. Mutations providing resistance to 2′-modified analogues have been identified more than a decade ago in the HCV RdRp gene [77]. The main resistance mutation is motif-B S282T (corresponding to S603 ZIKV), which provides a moderate resistance towards 2′-modified nucleotides [78]. The latter includes sofosbuvir (a prodrug transformed in the cell into 2′-deoxy-2′-fluoro-2′-C-methyluridine 5′-monophosphate, and later triphosphate) now widely used in the treatment of HCV [79]. So far, S603 of ZIKV RdRp is indeed included in the very few resistance and fidelity mutations that have been identified in the RdRp domain of *Flavivirus* NS5 [17,80,81]. Natural sensitivities to these analogues may well differ between HCV and flaviviruses such as DENV and ZIKV. Indeed, the composition of amino acids lining this pocket may well control its own conformational shaping upon binding the incoming nucleotide, as it has been shown for DENV and ZIKV NS5. The latter are less prone to be tricked by 2′-C-F- or 2′-C-Me-nucleotides than HCV RdRp [66].

Finally, if we surmise that (i) for *Flavivirus* RdRp, as for HCV RdRp [30], NTP-mediated repair (Figure 3) is involved in fidelity through mismatch excision; and (ii) that motif B potentially bears a role in translocation, then this could be another way by which motif B is involved in fidelity. Indeed, mutations in motif B could slow down translocation and increase the time of residency of a mismatch in the active site and, thus, its propensity to be excised by an NTP. This would have to be verified in the case of *Flavivirus* RdRps. In any case, motif B appears central for overall RNA synthesis fidelity, being involved both at the nucleotide probing and accommodation step and, potentially, in the mismatch–excision step.

### 3.3. Motif D

During catalysis where the 3′-hydroxyl is deprotonated to attack the phosphorus center, an event of proton-transfer to the leaving pyrophosphate must occur to ensure overall neutrality of the reaction. Several studies [63,82] proposed that a basic residue in motif D fulfills the role of a general acid donating a proton to the pyrophosphate during the rate-limiting transition state (Figure 1 step 3) of PV RdRp and also of other nucleic acid polymerases. Evidence was presented that PV RdRp residue K359 may act as the general acid. Structural studies, molecular dynamics simulations, and NMR studies proposed that the motif-D loop with K539 moves during catalysis [15] and changes from an open to a closed conformation for PV RdRp [40] (left panel of Figure 5C) and EV71 RdRp [41]. After catalysis, a conformational change occurs; and pyrophosphate has to be ejected from the active site in order to minimize pyrophosphorolysis and drive forward the polymerization reaction (Figure 1 steps 4 and 5). Motif D might be involved in these steps.

Motif D is the least conserved motif in positive-strand RdRps at the sequence and structure levels. It starts after a conserved α-helix and is largely made of structurally-diverse loops located at the bottom entrance of the NTP entry tunnel (Figure 5C). Sequence or structural alignment is difficult because of this diversity. Nevertheless, a Lys is found at a similar place in various positive-strand RNA virus RdRps (alignment Figure 4) even in DNA polymerases [82]. As an exception, HCV RdRp does not contain a Lys in motif D. For other polymerases it has been demonstrated that different basic amino acids (His, Arg) could donate a proton and thus function as a general acid. Note that the general acid is not absolutely essential but provides a 50-fold to 2000-fold rate enhancement, depending on the polymerase evaluated [82]. Interestingly, time-resolved crystallography of a DNA polymerase has evidenced a third metal ion, arriving after catalysis and stabilizing the pyrophosphate reaction intermediate [83]. It is not currently known if this mechanistic detail could apply for RNA polymerases as well.

In *Flavivirus* RdRps, there are three conserved residues in motif D giving a signature KxRK (Weblogo Figure 4). The last Lys in motif D (K691 in ZIKV) is appropriately located to close the active site and interact with the pyrophosphate (right panel of Figure 5C). The current conformation varies in the existing *Flavivirus* RdRp structures, especially for the side chain of the Lys. An adjustment would be necessary upon reaction as it was proposed with the structures of PV RdRp (Figure 5C and Table 1). Low-fidelity Q-β RdRp contains a Lys that might also interfere as a general acid although the loop and the Lys side chain conformation do not change between apo (Figure 5C right panel) and captured complex structures.

Several mutations in *Enterovirus* motif D have been reported to impact fidelity and provide viral mutator phenotypes, which are of interest in the design of attenuated vaccines [84,85]. Liu et al. [85] reported that a T362I change in motif D yielded a low fidelity PV RdRp. The authors propose that substitution alters the conformational equilibrium between open and closed states of PV RdRp. Mobility of the motif-D loop is certainly crucial in the fidelity process as also shown by McDonald et al. [84]. They report a higher fidelity CVB3 RdRp mutated in the motif-D loop (F364) where some mutant proteins presented motif D locked in the closed state. Since the motif-D loop is structurally very diverse, the corresponding residues of *Flavivirus* RdRps are difficult to pinpoint. Future experiments will have to show fidelity determinants in the *Flavivirus* motif-D loop (see Figure 4). 

The mechanism by which motif-D mutations affect fidelity remains elusive. It is tempting to speculate that an increased time-residency of the pyrophosphate in the active site might (positively or negatively) affect pyrophosphorolysis of a misinserted nucleotide, accounting for the change in fidelity. Alternatively, if reaction is slowed down, a slowed-down translocation of the primer may allow more time for occurrence of a nucleotide-excision repair such as the one described for HCV RdRp [30].

### 3.4. Motif E

Motif E consists of a β-hairpin situated between the palm and thumb subdomain. Whereas the structure is strictly conserved, there is only partial conservation at the sequence level for certain groups of RdRps. *Flavivirus* RdRps show a signature xFCSxHx7DGRx4PCRx (WebLogo Figure 4). The CSx_18_R is also found in *Flaviviridae* RdRps (see alignment Figure 4). In the existing structures of *Flaviviridae* RdRp motif E is localized near the priming loop. In BVDV RdRp two residues, conserved in *Flaviviridae* RdRps and corresponding to S712 and R731 in ZIKV RdRp, have been identified as being vital for de novo initiation and important for elongation efficiency [86]. Motif E has also been named the “primer-grip motif”. Indeed, the HCV RdRp elongation complex (e.g., PDB ID: 5WTA, [43]) shows that the residues corresponding to S712 and R731 contact the α-phosphates of priming nucleotide P−1 (just incorporated and translocated) and nucleotide P−2, respectively. Their positioning and, thus, the correct conformation of motif E might be important for the exact alignment of the 3′OH of the priming nucleotide −1. However, the impact of mutants within motif E on RdRp fidelity is unknown. Interestingly, the CVB3 RdRp mutant A372V just before motif E (corresponding to V708 in ZIKV RdRp) exhibits a change in fidelity [87].

### 3.5. Motif F

**Figure 6:**
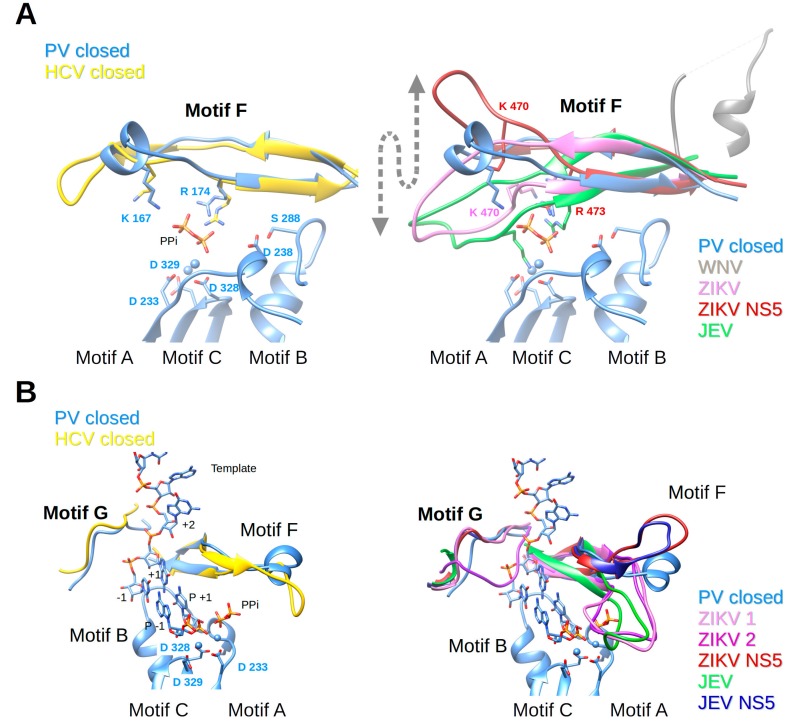
Motifs F and G of PV RdRp and HCV RdRp complexes (left panels) in comparison to motif conformations of *Flavivirus* RdRps (right panels). (**A**) Motif F; left panel: ribbon representation of PV RdRp (PV closed, PDB ID: 3OL7, blue) in complex with the template and product represented in sticks (colored according to atom type: C, blue; O, red; P, orange), and two Mg^2+^ ions as blue spheres, just after the incorporation of P+1. The formed pyrophosphate (PPi) is still present at the catalytic site. Only motif F is shown of HCV RdRp (HCV closed, see explanations in the legend of Figure 5B, PDB ID: 4WTA, gold). Selected amino acid side chains are shown and colored according to atom type. Right panel: PV closed (blue) as in the left panel superimposed to WNV RdRp (PDB ID: 2HFZ, grey, motif F only partial and in helical conformation, 90° to the catalytic conformation), ZIKV RdRp (PDB ID: 5UOC, orchid), ZIKV NS5 (PDB ID: 5U0B, dark red), JEV RdRp (PDB ID: 4MTP, green). The arrow indicates the significant flexibility of motif F in *Flavivirus* RdRps. (**B**) Motif G; left panel: as in (**A**), PV RdRp (PV closed, PDB ID: 3OL7, blue) with the template, product, and PPi. Only motifs G and F are shown of HCV RdRp (HCV closed, see explanations in the legend of Figure 5B, PDB ID: 4WTA, gold). Right panel: PV closed (blue) as in the left panel superimposed to ZIKV RdRp (ZIKV 1 and ZIKV 2, chains A and D, respectively, of PDB ID: 5UOC, orchid and purple, respectively), ZIKV NS5 (PDB ID: 5U0B, dark red), JEV RdRp (PDB ID: 4MTP, green, motif G only partial) and JEV NS5 (PDB ID: 4K6M, dark blue).

Nucleotides enter the polymerase active site through a tunnel whose ceiling is made of motif F. In most of the viral RdRp structures in a catalytic state, it starts with a conserved β-strand, then adopts diverse structures to finally end up forming another conserved β-strand. Sequence conservation within positive-strand RNA virus RdRps is limited to some positively-charged residues, which can be superimposed structurally (alignment Figure 4). The implication of motif F in fidelity especially concerns the interaction of these positive residues with the phosphate groups of the incoming NTP and the pyrophosphate leaving the active site. The open and closed structures of PV RdRp [40] proposed that the phosphates are probed and aligned by contacts with motif-F residues K167 (γ-phosphate) and R174 (α-phosphate). Conformational changes between these structures are restricted to side chain adjustments of K167 and R174. NMR methods support conformational changes in motif F during the reaction [88]. In Figure 6A (left panel) motif F of the closed complex structures of PV RdRp (PDB ID: 3OL7) and HCV RdRp (PDB ID: 5WTA) are shown with superimposed Lys and Arg residues.

Motif F in *Flavivirus* RdRps shows higher conservation at its beginning and end than in its central region (WebLogo Figure 4). Its signature is xYNxMGK(R/K)EKKxxxxGxAKGSRxTWxM. Structurally, the *Flavivirus* motif F seems to be extremely flexible in comparison to other RdRps. In most of the isolated RdRp domain structures (except for two apo ZIKV and JEV RdRp structures (PDB ID: 5U0C and 4MTP, respectively) and JEV RdRp in complex with GTP in a non-catalytic position (PDB ID: 4HDG)), motif F is only partially resolved. The end of the motif, which is present, adopts an alternative helical conformation (in Figure 6A (right panel) illustrated for WNV RdRp, PDB ID: 2HFZ) and is found in the place of motif G (see below). In DENV2 RdRp in complex with an inhibitor (PDB ID: 5K5M) motif F is also partially formed, but the end does adopt, as expected from other RdRp structures, a β-strand conformation forming the ceiling of the NTP tunnel. Concerning the full-length NS5 structures, in DENV3 NS5 (PDB IDs 4V0Q, 5CCV) motif F is also only partially resolved and adopts the same alternative conformation as in DENV3 or WNV RdRp domain structures. In contrast, in JEV and ZIKV NS5, the full motif F is present in a near-catalytic position. The different near-catalytic conformations of *Flavivirus* motif F are illustrated in Figure 6A (right panel) showing ZIKV RdRp and NS5 (PDB ID: 5U0C and 5U0B, respectively), JEV RdRp (PDB ID: 4MTP) and WNV RdRp (PDB ID: 2HFZ). They are compared to the “closed active site” complex structures of PV RdRp (PDB ID: 3OL7); for clarity only the leaving pyrophosphate group from the PV RdRp complex is shown. This structural alignment proposes that conserved ZIKV RdRp residues K470 and R473 are the ones contacting the phosphate groups of the incoming NTP in *Flavivirus* RdRps. While residue R473 seems to be in place (with some side chain changes) to probe and align the α-phosphate, K470 is displaced in the full-length NS5, and also in the isolated RdRp domain structures of ZIKV and JEV RdRps (see also Table 1). Especially, the tip of the ring finger adopts different positions due to direct contact with the MTase domain (ZIKV and JEV NS5) or due to other interactions with the index finger and the thumb subdomain (ZIKV and JEV RdRp). Neither of these positions seems to reflect the position of the fingertip during catalysis but they may show intermediate conformations relevant for replication. Interestingly, there is evidence that the beginning of motif F of *Flavivirus* RdRps is involved in promoter recognition for RNA synthesis, which is a key step for efficient initiation of RNA synthesis at the 3′ end of the RNA genome [89]. Pre-catalytic conformational changes of motif F seem indeed to be functionally relevant.

Concerning fidelity determining residues in motif F, Abdelnabi et al. [14] have shown that CVB3 RdRp bearing a K159R mutation (start of motif F corresponding to ZIKV RdRp residue K458, see Figure 4) shows a decrease in fidelity. There are obviously structural and biochemical determinants other than mere electrostatic determinants in motif F. Interestingly a recombinant virus is only viable with K159R and A239G, the latter being a motif-A low-fidelity variant [90]. Furthermore, mutation of FMDV RdRp residue V173 (corresponding to PV and CVB3 RdRp V168) in the tip of the ring finger altered fidelity [90,91]. The RdRp structure of the mutant shows that the mutation does not change the conformation of motif F, but causes long-range effects on the RNA template channel and the flexible loop of motif B. Due to the difference in fingertip conformations, corresponding *Flavivirus* motif-F residues cannot be proposed. Finally, motif-F mutant I176V in CVB3 RdRp shows slightly lower fidelity [18,90], whereas the corresponding V553I mutation in CoV MHV RdRp shows higher fidelity [92]. In BVDV RdRp (*Flaviviridae*, *Pestivirus*) corresponding mutation I287A (and R285A) also show altered fidelity [93]. In *Flavivirus* RdRps this putative fidelity determinant is an Ile (I475 In ZIKV RdRp). In conclusion, it would be interesting to mutate homotropic residues in *Flavivirus* RdRps and assay fidelity changes. Clearly, motif F is important for RdRp fidelity, being implicated in guiding both the nucleotide into the tunnel in order to be paired to the template base and aligned, and also in guiding the pyrophosphate out of the tunnel.

### 3.6. Motif G

Motif G is situated at the entrance of the RNA template tunnel. It is merely a structural motif without any sequence conservation in viral RdRps (alignment Figure 4). Figure 6B (left panel) shows how the motif is located near a kink in the phosphate backbone between template nucleotides +1 and +2 in the closed PV RdRp complex (PDB ID: 3OL7). Motif G in the HCV RdRp complex adopts a similar conformation.

*Flavivirus* RdRps show a motif-G signature xVxxxAAxGx (Weblogo Figure 4). Motif G also shows high flexibility in *Flavivirus* RdRps and, interestingly, this seems to be in concert with the folding of motif F. In several isolated RdRp structures without electron density for motif F, motif G is also not resolved (such as WNV RdRp [44], DENV3 RdRp [45,46] and several inhibitor complex structures [47,48,49], DENV2 [48], and most recently two ZIKV RdRp structures (PDB ID: 5U04 and 5WZ3 [51,52]). Interestingly, it is only partially resolved (Figure 6B right panel) in JEV RdRp (PDB ID: 4MTP [50]) where motif F is in place. Additionally, motif G is present in one ZIKV RdRp structure (PDB ID: 5U0C), but in two different conformations represented by chain A (ZIKV 1, canonical conformation) and D (ZIKV 2, displaced into the RNA template tunnel (Figure 6B right panel)). Note that in these structures motif F is in its place. In the JEV RdRp structure with GTP or ATP (PDB ID: 4HDG and 4HDH [50]) motif G is displaced into the RNA template channel similar to chain D of ZIKV RdRp (not shown in Figure 6). Motif G is also present in an isolated DENV3 structure with an inhibitor in the RNA template tunnel (PDB ID: 3VWS), which interacts with and displaces motif G into the RNA template tunnel (not shown in Figure 6). In JEV and ZIKV full-length NS5 motifs F and G are present with motif G adopting a very similar conformation to ZIKV RdRp chain A (Figure 6B right panel). It is, thus, plausible that the folding of motifs F and G is a concerted process and that motif-F folding precedes the folding of motif G.

Once in place motif G guides the RNA template into the tunnel (see RNA template of PV RdRp complex Figure 6B). One could, therefore, expect that it is implicated in fidelity because mutations in motif G could change the orientation of template residues +2 and +1 and so alter NTP recognition. One example for a *Flavivirus* RdRp is a mutation in a ribavirin-resistant RdRp of SLEV [67] corresponding to E416 in ZIKV RdRp. This residue is conserved as E/D/N in *Flavivirus* RdRps and located four residues after motif G on the surface of the protein. Likewise, a high-fidelity EV71 RdRp variant with mutation L123F is situated just after motif G [94]. Another example arguing that template positioning is decisive for fidelity was presented for FMDV RdRp where the mutation P44S (together with P169S) provided attenuation of the mutagenic effects of ribavirin. Whereas there is no structural effect of the P169S mutation, P44S positioned after the index finger influences the conformation of the latter. The index finger in *Enterovirus* RdRps forms a binding pocket for the +2 template base opposite to motif G [64]. In conclusion, the correct placement of template residues is expected to be associated with NTP or NTP analog recognition, alignment, and incorporation and, therefore, motif G, and possibly also the conformation of the index finger, are likely to be fidelity determinants.

### 3.7. Priming Loop

As mentioned above, the priming loop of *Flavivirus* RdRps is proposed to form a specific ATP binding site since de novo initiation on natural template always starts with A. It was shown that DENV2 RdRp has a strict preference for ATP in presence of Mg^2+^ ions, less pronounced in the presence of Mn^2+^ ions [61]. In the absence of relevant complex structures we do not know which residues constitute this ATP-binding site apart from H800 (ZIKV numbering) [61]. After de novo initiation the priming loop has to move out of the way, allowing the exit of the dsRNA product. Interestingly, Van Slyke et al. [17] reported two mutations in putative hinge positions of the priming loop of WNV RdRp, V793I and G806R corresponding to in ZIKV V790 and G803, which provide an increase in the fidelity of RNA synthesis. The priming loop during elongation mode is remote from the active site and the nucleotide selection pocket made of motifs A, B, D, and F. Thus, one can only speculate that when the priming loop is positioned properly for elongation, these mutations promote a slightly altered positioning of the primer/template RNA that translates, via long-range effects, into an increase of selectivity of the incoming nucleotide.

## 4. Conclusions

**Figure 7:**
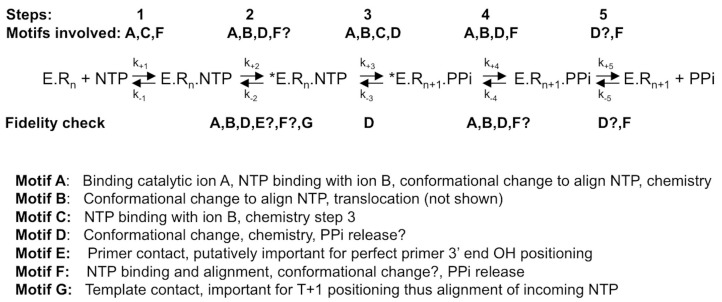
Current model of the involvement of catalytic and structural motifs of *Flavivirus* RdRps in NTP incorporation into RNA and RNA synthesis fidelity. Above the reaction scheme catalytic steps (explained in Figure 1, lower scheme) are given and the involvement of the motifs in each step. Below the putative involvement of the motifs in RNA synthesis fidelity is given based on evidence or assumptions explained in the text. For explanations of the reaction scheme, see the legend of Figure 1.

A great variety of mechanisms are involved in the selection of correct versus incorrect nucleotides at the *Flavivirus* polymerase active site, and the structural basis of this selection process waits to be deciphered with precision. The conservation of the viral RdRp scaffold and motifs around the active site provides a useful frame to envisage testing individual and coordinated actions of amino acids involved in NTP selection. In Figure 7, we present a summary of the putative involvement of the *Flavivirus* motifs in the RNA synthesis steps outlined in Figure 2 and in RdRp fidelity. Proposals for individual point mutations are made in each motif section. There is little doubt that additional mechanisms than those mentioned here will be identified in the near future, perhaps with their associated enigmas before novel techniques and concepts cast light on this fascinating process. Structural biology, molecular dynamics, and modeling techniques will probably play an important role in the understanding of results still challenging a sound interpretation. For example: how do subtle conformational changes induced by divalent metal-binding (Mg^2+^, Mn^2+^, …) distort the active site?; how do other domains, such as the MTase domain, affect the fidelity of the RdRp [17]?; how do amino acid changes remote from the active site transduce alterations of substrate selection though long-range interactions?

Drawing from AZT-resistance by HIV reverse transcriptase and the seminal paper of Jin et al. [30] describing a related process of 3′-nucleotide excision by the HCV RdRp, it is highly probable that similar post-nucleotide incorporation mechanisms exist for a number of viral RdRps. Not only their contribution to the overall fidelity process is unknown, but also the molecular events involved remain largely uncharacterized at the biochemical and structural levels. Likewise, the pyrophosphorolysis process and its relation to translocation and fidelity remain a wide-open field. Understanding the fidelity of viral RNA and genome synthesis will have a great impact in the understanding of how viruses remain the most abundant, adaptable, and successful form of life.

## Figures and Tables

**Table 1:** Analysis of active site conformation of selected plus strand RNA virus RdRp structures.

Virus	RdRp or Complex	Active Site	PDB		Motif			
			ID	Resolution (Å)	Name	Conformation	Residue	Position
PV	Complex RdRp.P/T after reaction and translocation	Open	3OL6	2.5	A	β-strand displaced	D233	Out
							D238	In
					B	Loop in place	S288	Out
					D	Loop displaced	K359	Out
					F	In place	K167	Out
							R174	Out
	Complex RdRp.P/T after reaction	Closed	3OL7	2.7	A	β-strand in place	D233	In
							D238	Out
					B	Loop in place	S288	In
					D	Loop in place	K359	In place
					F	In place	K167	In place
							R174	In place
HCV	Apo RdRp	Open	2XI2	1.8	A	β-strand displaced	D220	Out
							D225	In
					B	Loop in place	S282	Out
					D	Loop open	No K/H/R in motif D	
					F	In place	K155	Out
							R158	Out
	Complex RdRp.P/T.NDP “before” reaction	Closed	4WTA	2.8	A	β-strand in place	D233	In
							D238	Out
					B	Loop in place	S288	In
					D	Loop closer	No K/H/R in motif D	
					F	In place	K155	Out
							R158	In place
Q-β	Apo RdRp	Partially open	3AGP	2.8	A	β-strand in place	D968	In
							D974	Further away
					B	Loop in place	M1017	Out
					D	Loop in place	K1080	Out
					“F”	In place	K908	In place
							R914	In place
	Complex RdRp.P/T after reaction	Partially open	3AVV	2.6	A	β-strand in place	D968	In
							D974	Further away
					B	Loop in place	M1017	Out
					D	Loop closer	K1080	In
					“F”	In place	K908	In place
							R914	In place
ZIKV	Apo RdRp	Open	5U0C chains A/D	3.0	A	No β-strand, displaced	D535	In displaced
							D540	In
					B	Loop in place/displaced	S603	Out/in
					D	Loop	K691	In
					F	Partially in place	K470	Out
							R473	Out
JEV	Apo NS5	Open	4K6M	2.6	A	No β-strand, displaced	D536	In displaced
							D541	In
					B	Loop displaced	S604	Out
					D	Loop	K694	In
					F	Partially in place	K471	Displaced
							R474	In place
DENV3	Apo NS5	Open	5CCV/4V0Q	2.3	A	No β-strand, displaced	D533	In displaced
							D538	In
					B	Loop in place/displaced	S600	Out/in
					D	Loop	K689	Out
					F	Totally displaced	K471	Not present
							R474	Displaced
WNV	Apo RdRp	Open	2HFZ	3.0	A	No β-strand, displaced	D536	Out
							D541	In
					B	Loop largely displaced	S604	Out
					D	Loop	K689	Out
					F	Totally displaced	K471	Not present
							R474	Displaced

Open or closed complex structures of PV and HCV RdRp were analyzed for the conformation of motifs A, B, D, F, and side chain positions of certain residues implicated in NTP probing, alignment, and catalysis. Conformation of complex and apo-structures of bacteriophage Q-β and selected Flavivirus RdRps were analyzed and described in comparison. PDB ID = Protein Data Bank Identification code, RdRp.P/T = binary complex RdRp primer/template. For the ZIKV RdRp structure 5U0C two chains, A and D, were considered, which show differences in motif B (loop in place and displaced, respectively, and S603 out and in, respectively). For DENV3 NS5 we listed two structures, 5CCV (deleted priming loop) and 4V0Q; they present likewise differences in motif B.

## Supplementary Materials

The following are available online at http://www.mdpi.com/1999-4915/10/2/59/s1, Video S1: Spatial Arrangement of RdRp Motifs; Video S2: RdRp Motifs in the Mechanism of NTP Incorporation.
